# Diblock, Triblock and Cyclic Amphiphilic Copolymers with CO_2_ Switchability: Effects of Topology

**DOI:** 10.3390/polym12040984

**Published:** 2020-04-24

**Authors:** Yuting Jiang, Tong Zhang, Zheng Yi, Yixiu Han, Xin Su, Yujun Feng

**Affiliations:** 1 Polymer Research Institute, State Key Laboratory of Polymer Materials Engineering, Sichuan University, Chengdu 610065, China; jiangyt99@126.com (Y.J.); zhangtong2099@163.com (T.Z.); 2 The Second Research Institute of Civil Aviation Administration of China, Chengdu 610041, China; yizheng@caacsri.com (Z.Y.); hanyixiu@caacsri.com (Y.H.)

**Keywords:** CO_2_, cyclic copolymer, amphiphilic copolymer, CO_2_ sensitivity

## Abstract

The combination of topology and CO_2_ switchability could provide new options for amphiphilic copolymers. Cyclic molecules supply novel topologies, and CO_2_ switching provides stimulus responsiveness. Cyclic poly(2–(diethylamino)ethyl methacrylate)–b–poly(ethylene oxide) and their corresponding block copolymers were prepared from poly(ethylene oxide) and 2–(diethylamino)ethyl methacrylate via atom transfer radical polymerization and Keck allylation with a Hoveyda–Grubbs catalyst. Changes in conductivity, surface activity, and hydrodynamic size were examined to illustrate the switchability of the produced amphiphilic copolymers upon contact with CO_2_ in the presence of water. The reversible emulsification and switchable viscosity behaviors of the copolymers were also demonstrated.

## 1. Introduction

CO_2_ has promising prospects as a responsive trigger due to its vital advantages of low cost, environmental friendliness, and wide availability. Amidines and amines are CO_2_–responsive molecules [1,2,3,4] that can react with dissolved CO_2_ in water and generate charged amidinium bicarbonate or ammonium salts. These reactions can be reversed by Ar or N_2_ bubbling. Because this regulated process involves only CO_2_ and other inert gases if no other impurity is introduced [5], it may have various applications in a wide range of important industries. Many different responsive approaches in which CO_2_ is used as a trigger in the form of surfactants [5], solvents [6], organic salts [7], and polymer emulsions [8] have been reported to achieve this goal.

Aqueous solutions using polymeric surfactants with adjustable surface activities are also attractive. Small–molecule surfactants appeared first and Jessop’s research group successfully prepared a series of intelligent CO_2_ switchable surfactants by using CO_2_ as a triggering substance to realize CO_2_–responsive surface activity [5,9]. Our group applied tertiary amines with an ultralong hydrophobic chain and discovered another method wherein CO_2_ could be utilized to adjust the surface activities of aqueous solutions [10,11,12]. One of the key advantages of polymeric surfactants over traditional surfactants is the high level of tailoring that can be done with the former to afford optimal stabilization properties. Lu et al. developed CO_2_–switchable polymeric surfactants synthesized with 2–(dimethylamino)ethyl methacrylate and the hydrophilic monomer acrylamide [13]. The polymeric surfactants displayed good performance for emulsifying heavy oil emulsions. Another merit of polymeric surfactants is that their chemical structure, composition, molecular weight, and topology can be easily modified [14]. We previously reported CO_2_–switchable multicompartment micelles with a segregated corona that self–assembles from a linear ABC triblock copolymer. This triblock copolymer aggregates into uniform spherical micelles in the absence of CO_2_ and can be switched into micelles with a segregated corona in the presence of CO_2_ [15].

Adjustments in surface activity are based on the molecular structure of surfactants, and topological modification is a powerful approach to construct functional polymers with molecular–level precision [16]. However, the molecular topology of CO_2_–switchable polymeric surfactants has been insufficiently researched, and investigations on block copolymers mainly focus on their molecular weight and dispersity. Developing an alternative method to modify the molecular topologies of CO_2_–switchable polymeric surfactants is a challenging topic in polymer chemistry. Polymeric surfactants can be categorized as diblock or triblock copolymers according to the number of their hydrophobic and hydrophilic segments. In nature, cyclic lipids are found in the cell membranes of organisms living in high–temperature environments; some examples of these organisms include thermophilic bacteria in springs and submarine volcanos [16,17,18]. Inspired by this phenomenon, researchers have synthesized an amphiphilic polymer with a cyclic structure that could provide a new approach for obtaining CO_2_–switchable polymeric surfactants. However, because the topology of CO_2_–switchable copolymeric surfactants is rarely reported, choices for fabricating CO_2_–sensitive copolymers are limited.

In this study, we present an example of a CO_2_–switchable polymeric surfactant showing significant topological effects and including diblock, triblock, and cyclic amphiphilic copolymers. The influences of chemical structure and segment composition on the topological effect and tendency of the CO_2_ response are investigated by evaluating the properties of the self–assembled micelles. Specifically, poly(ethylene oxide) and 2–(diethylamino)ethyl methacrylate (DEAEMA), acting as the hydrophilic and CO_2_–switchable hydrophobic sections, respectively, are used to synthesize cyclic poly(DEAEMA)–b–poly(ethylene oxide) (copolymer 1), diblock linear polymer poly(DEAEMA)–b–poly(ethylene oxide) (copolymer 2), and triblock linear polymer poly(DEAEMA)–b–poly(ethylene oxide)–b–poly(DEAEMA) (copolymer 3). The relevant properties (i.e., surface activity) of micelles of the three different surfactants are then analyzed and compared.

## 2. Materials and Methods

### 2.1. Materials

DEAEMA, 2–bromoisobutyryl bromide, poly(ethylene oxide) (M_n_ = 2000), CuBr, 4,4′–dinonyl–2,2′–dipyridyl (dNbpy), allyltributyl stannane, and first–generation Grubbs catalyst were purchased from Sigma–Aldrich (St. Louis, MO, USA) and used as received. The poly(ethylene oxide) macroinitiator with 2–bromoisobutyryl terminal groups was prepared through esterification of poly(ethylene oxide) (Mn = 2000) or poly(ethylene glycol) methyl ether (M_n_ = 2000) with 2–bromoisobutyryl bromide according to a previously reported procedure [16].

### 2.2. Synthesis of Amphiphilic Linear Poly(DEAEMA)–b–Poly(Ethylene Oxide)–b–Poly(DEAEMA) (Copolymer 3)

The triblock copolymer was prepared via atom transfer radical polymerization (ATRP) and Keck allylation in accordance with a previously reported procedure [16]. The poly(ethylene oxide) macroinitiator (400 mg, 0.15 mmol), DEAEMA (420 mg, 23 mmol), dNbpy (30 mg, 73 μmol), and CuBr (10 mg, 70 μmol) were placed in a test tube. The mixture was degassed through freeze–pump–thaw cycling, and the test tube was sealed under vacuum. The resulting suspension was stirred at 110 °C for 1 h, cooled with liquid N_2_, and allowed to warm to 20 °C. Allyltributyl stannane (150 mg, 0.45 mmol) was added to the reaction mixture, and the resulting suspension was degassed through freeze–pump–thaw cycling. The test tube was sealed under vacuum, and the suspension was stirred at 110 °C for another 12 h. The reaction mixture was diluted with acetone and filtered through a plug of alumina. The eluent was concentrated and reprecipitated in *n*–hexane to allow the isolation of the product as a colorless waxy solid (448 mg) in 71% yield.

### 2.3. Synthesis of Amphiphilic Cyclic Poly(DEAEMA)–b–Poly(Ethylene Oxide) (Copolymer 1) 

First–generation Grubbs catalyst (30 mg, 36 μmol) was added to a CH_2_Cl_2_ solution of **3** (0.12 mM, 100 mL), and the resulting solution was refluxed for 48 h. The reaction mixture was concentrated and filtered through a plug of alumina. The eluent was concentrated and reprecipitated in cold *n*-–hexane to allow the isolation of polymer 1 as a colorless waxy solid (29 mg) in 56% yield. 

### 2.4. Synthesis of Amphiphilic Linear Poly(DEAEMA)–b–Poly(Ethylene Oxide) (Copolymer 2)

Diblock copolymer 2 was prepared via the same procedures applied to obtain polymer 3 except that a different macroinitiator was used. The poly(ethylene oxide) macroinitiator made from poly(ethylene glycol) methyl ether (M_n_ = 2000) (400 mg, 0.15 mmol), DEAEMA (420 mg, 23 mmol), dNbpy (30 mg, 73 μmol), and CuBr (10 mg, 70 μmol) were placed in a test tube. The mixture was degassed through freeze–pump–thaw cycling, and the test tube was sealed under vacuum. The resulting suspension was stirred at 110 °C for 1 h, cooled with liquid N_2_, and allowed to warm to 20 °C. Allyltributyl stannane (150 mg, 0.45 mmol) was added to the reaction mixture, and the resulting suspension was degassed through freeze–pump–thaw cycling. The test tube was sealed under vacuum, and the suspension was stirred at 110 °C for another 12 h. The reaction mixture was diluted with acetone and filtered through a plug of alumina. The eluent was concentrated and reprecipitated in *n*–hexane to allow the isolation of the product as a colorless waxy solid (524 mg) in 78% yield.

To test the transformation of amine group in the presence of CO_2_, the protonated copolymer 2 was prepared: copolymer 2 was dissolved in chloroform, then tiny amount of water was added into the solution with the sparging of CO_2_. After that, precipitation appeared, which was the protonated copolymer 2.

### 2.5. Characterization

Nuclear magnetic resonance (NMR) spectroscopy: ^1^H NMR spectra were obtained on a Bruker Avance–II 400 MHz NMR spectrometer (Billerica, MA, USA).

Gel permeation chromatography (GPC): GPC measurements (Shodex 3000 GPC system with an HPLC pump, Santa Clara, CA, USA) were conducted at 40 °C to measure the molecular weight of samples. The GPC system was equipped with a Waters separation module, including four polystyrene columns with pore sizes of 100, 500, 103, and 104 Å, and a differential refraction detector. Tetrahydrofuran was used as the eluent with a flowrate of 0.3 mL/min. Molecular weights were determined by using polystyrene standards in the range of 870,000−875,000 g/mol.

Surface tension: Surface tension at different concentrations was measured and recorded using a Krüss K100 full–automatic surface tension analyzer (Hamburg, Germany). When the standard deviation of five successive measuring points was less than 0.05 mN/m, the tension was defined as the equilibrium surface tension of the measured solution.

Hydrodynamic size: The hydrodynamic sizes of the copolymers were determined with a Malvern Zetasizer Nano ZS instrument (Malvern, UK). Impurities in the tested samples were removed with a 0.8 μm probe filter, the experimental temperature was set to 25 °C, and each sample was tested at least three times.

Conductivity: Conductivity was measured using a DDS–11A conductometer with a DJS–1C platinum electrode (Shanghai, China).

TEM: Transmission electron microscopy (TEM) visualization was conducted on a Hitachi H600 electron microscope (Tokyo, Japan).

Transmittance: A UV–Vis spectrophotometer (Mapada UV–6100, Shanghai, China) equipped with dual channels for detecting the reference deionized water and sample solutions was utilized for all measurements at 25 °C. Each sample solution was measured in triplicate to obtain average values.

Viscosity: Viscosity was measured on an MCR 302 (Anton Paar, Graz, Austria) rotational rheometer with a Searle–type concentric cylinder geometry (CC27), and the shear rate was set to 10/s. Prior to the measurements, sample solutions were equilibrated at 25 °C for 10 min with a Peltier temperature control device.

## 3. Results and Discussion

**Scheme 1:**
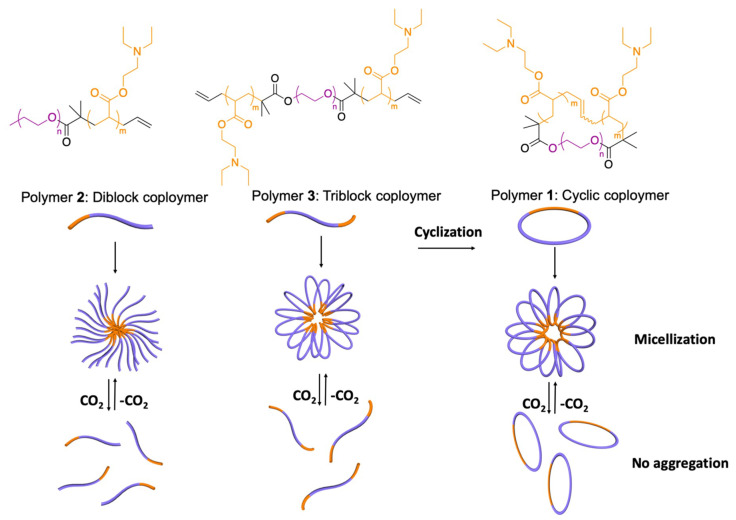
Chemical structures of polymer 1 as a cyclic amphiphilic block copolymer, 2 as a diblock copolymer, and 3 as a triblock copolymer. Schematic representation of the formation of micelles controlled by CO_2_.

**Figure 1:**
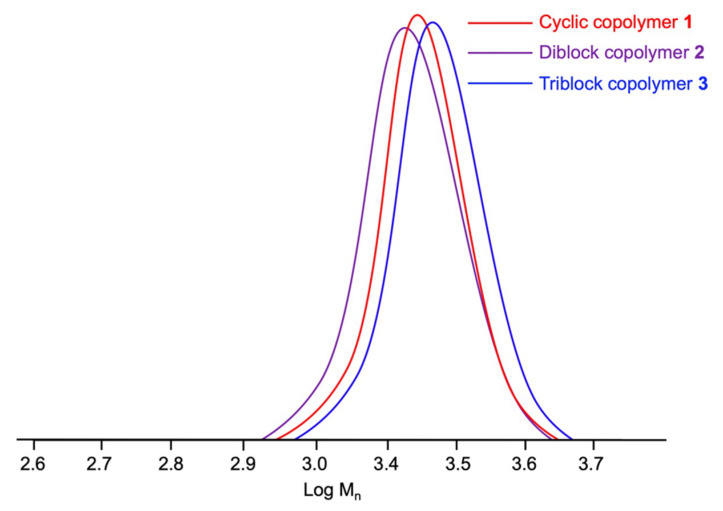
Gel permeation chromatography (GPC) traces of copolymers **1**, **2**, and **3.**

Linear triblock amphiphilic polymers containing different blocks, as well as the corresponding cyclic copolymer **1** (Scheme 1), were synthesized and characterized according to previously reported methods [16,19]. Copolymer 2 and copolymer 3 were prepared through ATRP [20] and Keck allylation [21]. Using copolymer 3 as a precursor, copolymer 1 was synthesized via cyclization triggered by metathesis polymerization with a second–generation Hoveyda–Grubbs catalyst [22] in DMSO. ^1^H NMR (Figures S1–S4) confirmed that propylene units were converted into internal olefin units through metathesis polymer cyclization. GPC revealed a decrease in hydrodynamic volume (Figure 1). The molecular weight of polymer 1 was lower than that of its precursor polymer 3, as shown in Table 1, thus indicating that the former presentrf a cyclic structure. After treatment of water and CO_2_, it was found that copolymer 2 had bicarbonate, proving that the polymer can be protonated by CO_2_ in the presence of water.

### 3.1. CO_2_ Switchability

**Figure 2:**
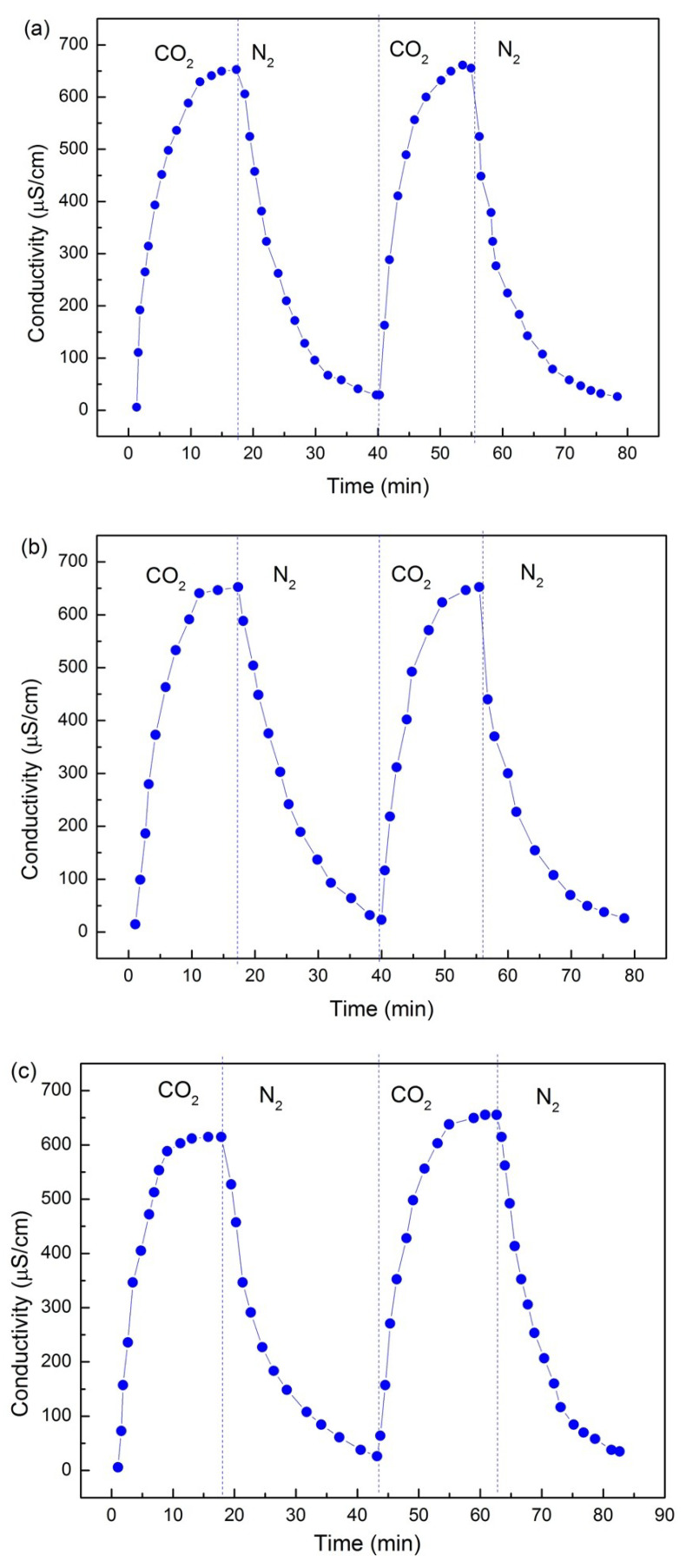
Conductivity of aqueous solutions of polymers **1** (**a**), 2 (**b**), and 3 (**c**) (0.2 wt%) as a function of time during two cycles of CO_2_/N_2_ sparging at 25 ± 0.5 °C.

Given that copolymers 1, 2, and 3 had a tertiary amine as their switchable hydrophobic segment and PEO as their permanent hydrophilic component, the three block polymers presented switchable behaviors that could be controlled by CO_2_. Determining changes in conductivity ratio is the most classic and effective method to verify the reversibility and repeatability of CO_2_/N_2_ switching (Figure 2). When polymers remained in neutral form in the absence of CO_2_ in an aqueous solution, the conductivity ratio of the solution was nearly zero. By contrast, when polymers reacted with CO_2_ and form cations in water, a large number of bicarbonate ions were distributed in the solution and the conductivity ratio increased drastically.

The experiments showed that the initial form of the polymer in the solution was a neutral nonionic compound; thus, its conductivity ratio is consistent with that of pure water. However, the conductivity of the solution in its initial state was slightly higher than zero, likely because of the presence of tertiary amines in the copolymers. Continuous insertion of CO_2_ led to the reaction of the polymers with CO_2_ and subsequent formation of quaternary ammonium bicarbonates, thus resulting in a rapid increase in conductivity [4]. When conversion was completed, the increase rate of the conductivity ratio gradually decreased and stabilized. As N_2_ bubbling continued, the bicarbonate salt gradually decomposed into a neutral nonionic compound, resulting in a gradual loss of conductivity. The conductivity of the solution eventually decreased to the initial value and gradually stabilized. The final conductivity measured was slightly higher than the initial value because of the incomplete reversible conversion of oxycarbonate. The conductivity experiments confirm the CO_2_/N_2_ responsiveness of the polymers and the repeatability of their switching behavior. 

### 3.2. Surface Tension

**Figure 3:**
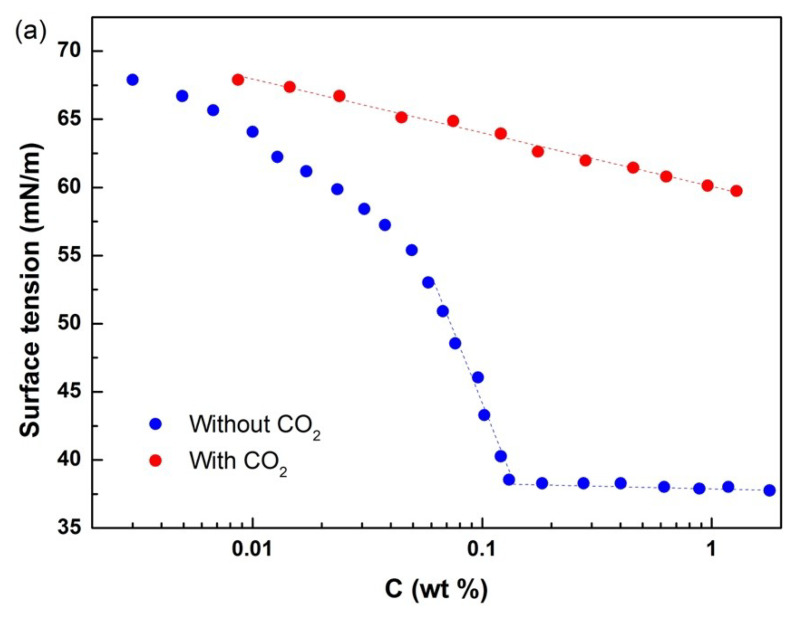

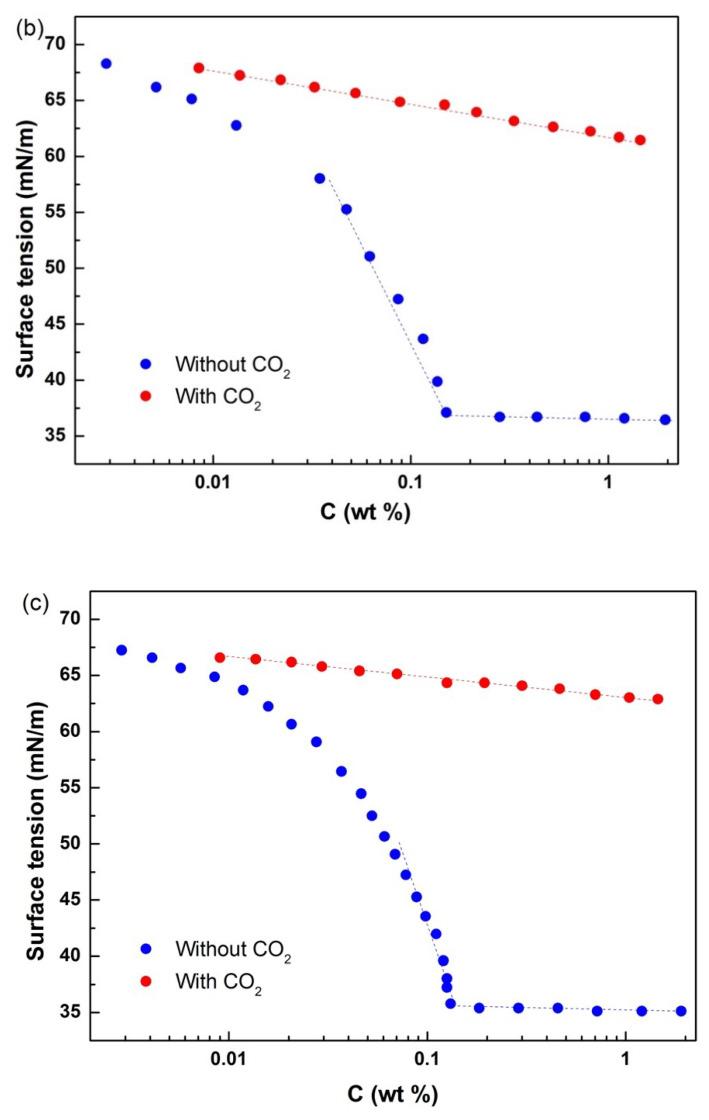
Surface tension of aqueous solutions containing copolymers 1 (**a**), 2 (**b**), and 3 (**c**) in the presence of air and CO_2_ at 25 ± 0.5 °C.

The conductivity test showed that the poly–DEAEMA segment of block polymers in the aqueous solution could be reversibly modified by using CO_2_ and that copolymers 1, 2, and 3 could exhibit reversible surface activity under the control of CO_2_. Figure 3 presents the surface tension and critical micellar concentration (CMC) of the copolymers. When the three copolymers were dispersed in aqueous solution without CO_2_, they behaved as normal surfactants. After CO_2_ bubbling, the whole polymers became hydrophilic due to protonation of tertiary amines in their hydrophobic segments. Therefore, the polymers lacked a CMC in the presence of CO_2_. When CO_2_ was expelled from the solution by using N_2_, the original properties of the polymers were restored, the micelles were regenerated, and surface activity returned to its original level with CMC. As the charged copolymers were converted into a neutral form, the ionic segment of DEAEMA became neutral. Therefore, the aqueous solution containing polymer 1 exhibited switchable surface tension behavior under the influence of CO_2_. The surface tension of the cyclic diblock copolymer was compared with those of the linear diblock and triblock copolymers, as shown in Table 1. The results showed that the three copolymers have similar surface tension data, thereby indicating that they had similar micelle–formation and assembly behaviors on water/air surfaces.

**Figure 4:**
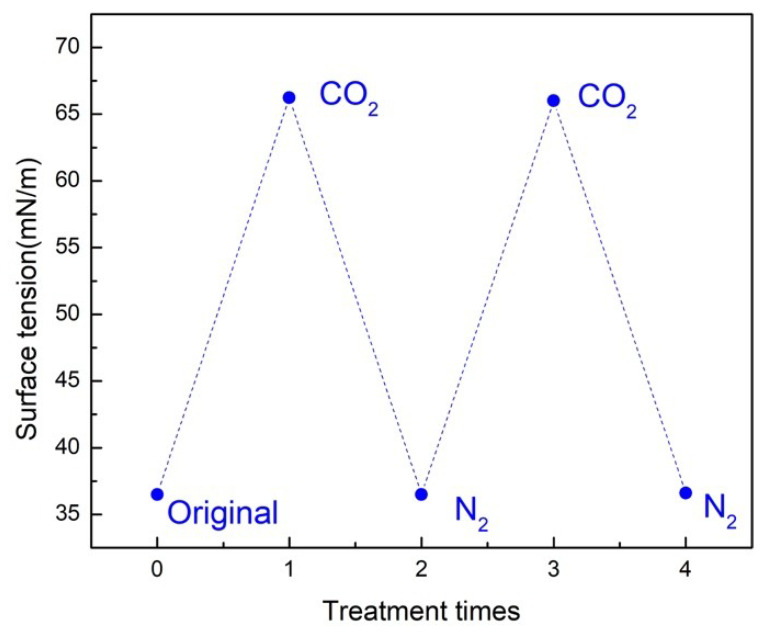
Changes in surface tension of an aqueous solution of copolymer 1 (0.2 wt%) as a function of treatment time under alternating two–cycle CO_2_/N_2_ bubbling.

CO_2_ can be a good trigger to change the surface tension of aqueous solutions with the three copolymers. In addition, the response to surface tension is reversible. As an example, the surface tension of an aqueous solution of polymer 1 during two cycles of alternating CO_2_/N_2_ bubbling was measured at 25 ± 0.5 °C (Figure 4). N_2_ bubbling was required to remove CO_2_ from the aqueous solution and subsequently neutralize carbamate. Figure 3 displays fluctuations in surface tension and pH of the aqueous solution containing copolymer 1 (0.2 wt%) during two cycles of alternating CO_2_/N_2_ bubbling. Surface tension decreased from 34.9 mN/m to 31.0 mN/m after CO_2_ bubbling for 20 min. pH fluctuated from 7.2 to 5.6 during alternating CO_2_/N_2_ bubbling. The initial value was reached after bubbling N_2_ into the aqueous solution.

**Figure 5:**
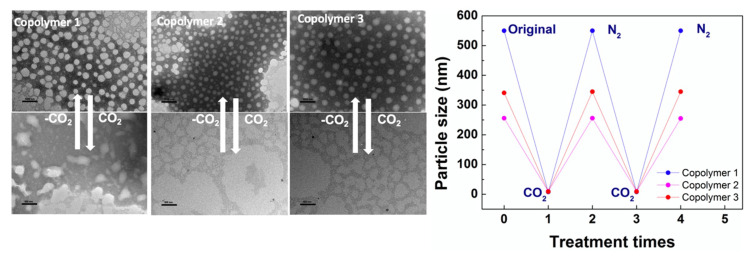
The TEM images (left) and change of hydrodynamic size (right) of the copolymers 1, 2 and 3 (0.2 wt%) in the presence or absence of CO_2_. The scale bars of TEM images in the absence CO_2_ (upper) are 1000 nm and those in the presence of CO_2_ (lower) are 100 nm.

Changes in effective diameter confirm the switchability of copolymer 1 under a CO_2_/N_2_ atmosphere. The concentration of copolymer 1 was determined to be 0.2 wt%. The results indicated that the diameters of the surfactants are affected by the atmosphere. Prior to CO_2_ sparging, polymer molecules tended to aggregate, forming micellar structures with large hydrodynamic sizes. During bubbling with CO_2_, the micellar structure was diminished because of the effect of electrostatic repulsion, resulting in small hydrodynamic sizes (Figure 5, right plot). The observed results prove that the obtained copolymer 1, a cyclic amphiphilic copolymer, could be reversibly converted into micelles via self–assembly in water. In the presence of CO_2_, copolymers 1, 2 and 3 did not form micelles; In the absence of CO_2_, the particle size (546 nm) of 1 was the largest among the three polymers. The possible reason is that cyclic structure of copolymer 1 promotes the formation of micelles and the increases the aggregate number of micelles.

### 3.3. Switchable Emulsion

Emulsification is an effective approach to reduce the viscosity of crude oil. If CO_2_ can trigger the reversible conversion of crude oil emulsions, the problems encountered in the process of crude oil transport through pipelines may be addressed. Specifically, crude oil emulsions with low viscosity can be prepared and water can be separated from crude oil after removing CO_2_ from or adding CO_2_ to crude oil emulsions after transportation.

**Figure 6:**
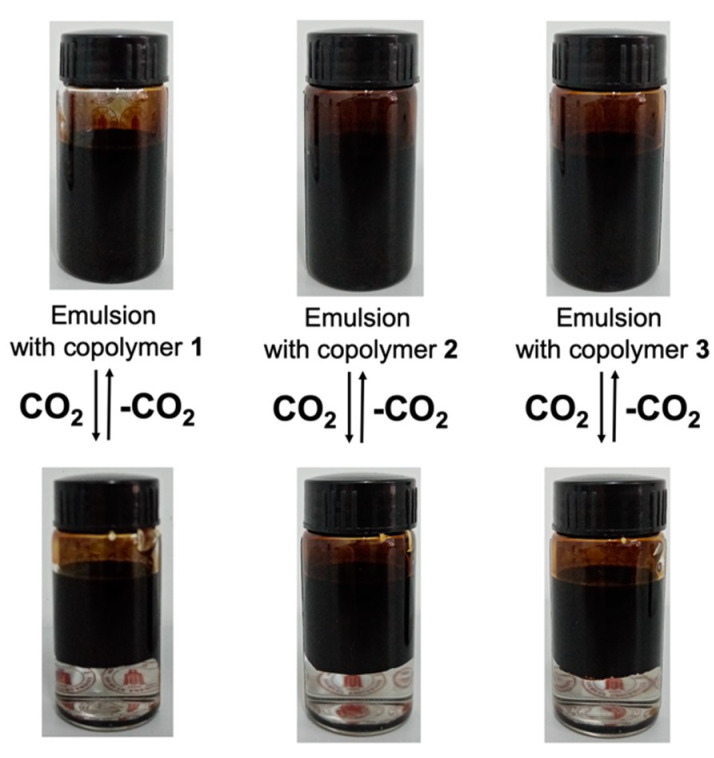
Reversible stability of crude oil emulsions and aqueous solutions. The aqueous solutions (5 mL) contained polymers 1, 2, and 3 (0.4 wt%). The original emulsion (top) was stable. Phase separation occurred when CO_2_ was introduced (bottom). A stable emulsion was regenerated after N_2_ spraying and shaking by hand.

Investigation of the CO_2_ responsiveness of the three copolymers indicated that the modification approach is also feasible for emulsions containing the copolymers. A crude oil–in–water emulsion (weight ratio, 0.8:1) was prepared using the copolymers (0.4 wt%) to confirm the effect of the copolymers on emulsion stability. Under this condition, the neutral copolymers 1, 2, and 3 behaved as impressive emulsifiers (Figure 6). Given the diffused electric double layer surrounding the milky droplets, an inner layer consisting of polymer 1 adsorbed on the surfaces of the droplets and the outer layer involved Na ions, which are attracted by surface charges. The neutral polymer 1 had no adverse effect on the electric double layer in the absence of CO_2_. Figure 4 shows that crude oil stably existed in the aqueous emulsion for more than 36 h after 20 min of CO_2_ bubbling. However, in the presence of CO_2_, tertiary amines in polymer 1 reacted with CO_2_ and generated polymeric bicarbonate salts. Therefore, polymer 1 lost its surfactant capability. Without the assistance of a surfactant, the stability of the emulsion drastically decreased as the emulsion droplets coalesced within 20 min after introducing CO_2_ to polymer 1 (Figure 4). The two phases became completely apparent after another 20 min. However, when N_2_ was sprayed onto a mixture containing crude oil/water phases to remove CO_2_, the tertiary amine of polymer 1 was converted back into its neutral form. Then, the mixture was stirred to prepare a stable emulsion. Alternating CO_2_/N_2_ bubbling could be used to convert emulsions into two phases. This conversion process triggers the behavior of the emulsion phase in the reservoir via the injection of CO_2_. Therefore, the emulsification of transferable crude oil by adding and removing CO_2_ to decrease or improve the stability of the emulsion of polymer 1 is possible. The results showed no differences among the performances of the three copolymers for reversibly emulsifying crude oil under the control of CO_2_.

### 3.4. Switchable Viscosity

**Figure 7:**
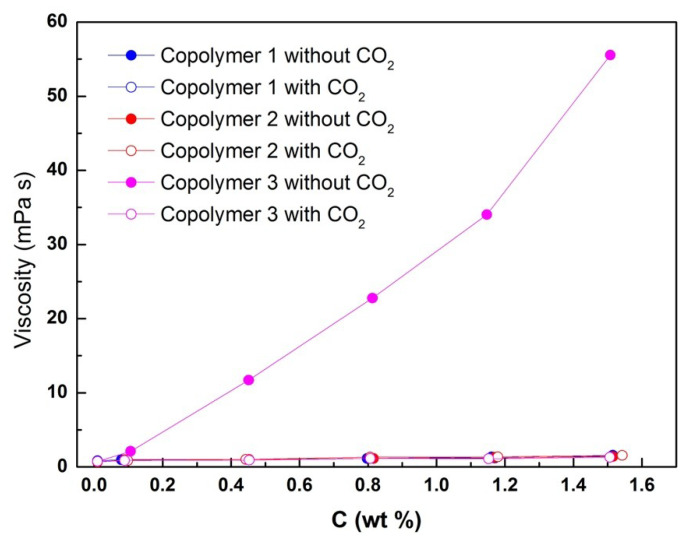
Viscosity of aqueous solutions containing copolymers 1, 2, and 3 in the presence of air and CO_2_ at 25 ± 0.5 °C.

The hydrophobic component of copolymers 1, 2, and 3 could be reversibly switched so that the materials become hydrophilic by CO_2_ addition, and using CO_2_ to control viscosity would be possible [23]. Figure 7 shows the viscosities of aqueous solutions containing copolymers 1, 2, and 3 at different concentrations in the absence or presence of CO_2_. The results showed that copolymer 3 had the highest viscosity among the samples in the absence of CO_2_. The viscosity of copolymer 3 likely increased because this copolymer, as a triblock polymer, can achieve cross–micellar connections through its two hydrophobic segments, thus forming a network structure at the microscopic level. The viscosity of copolymer 3 was CO_2_–responsive and diminished by addition of CO_2_. The viscosity of copolymer 3 is only slightly higher than that of pure water at 25 °C. Viscosity decreased because the conversion of the hydrophobic chain segments into hydrophilic cationic chains with bicarbonate counterions results in the breakup of hydrophobic associations. Viscosity measurements were subsequently carried out after bubbling N_2_ through the solution to remove CO_2_ and confirm that the switch was reversible. After 50 min, the viscosity recovered to its initial value.

### 3.5. Thermal Stability

**Figure 8:**
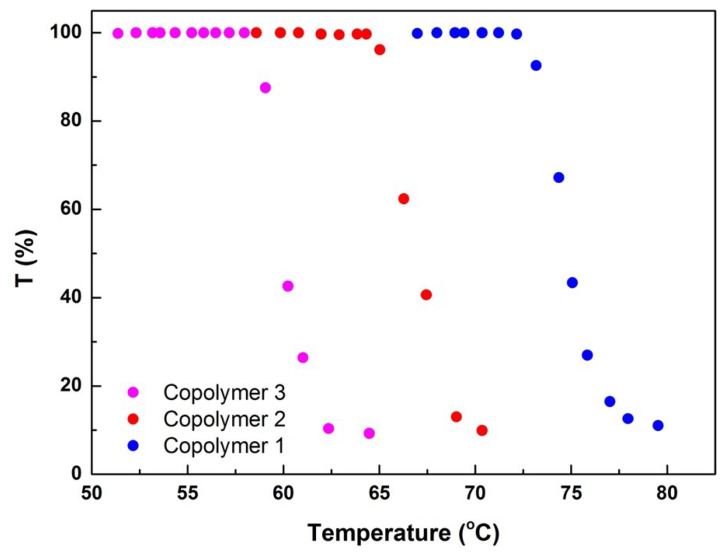
Temperature–dependent turbidity measurements of aqueous solutions containing copolymers 1, 2, and 3 (C, 1.0 wt%).

Given that all three copolymers contained PEO strands, they were expected to have cloud points, which means their aqueous solutions should have become cloudy when the temperature was increased to a certain extent. Aqueous solutions (1.0 wt%) of the copolymers were tested for light transmission at different temperatures to study the effects of topological structures on the thermal stability of the materials. Figure 8 reveals that the cloud point of copolymer 3 was 60 °C and that of copolymer 2 was 66 °C. Among the samples, cyclic copolymer 1 had the highest cloud point of 75 °C. The ring–shaped and linear assemblies exhibited significantly different thermal stabilities. The lower thermal stability of the linear triblock composition is attributed to intermicellar bridging caused by the combination of a suspended polymer chain and dehydration, which results in reunion at a low temperature [16]. Light transmission was not measured under CO_2_ because CO_2_ does not stably exist in aqueous solutions at temperatures above 60 °C [6,7].

## 4. Conclusions

Three types of copolymers with similar molecular weights and different topologies were prepared. These copolymers included a new cyclic poly(DEAEMA)–b–poly(ethylene oxide) (polymer **1**), which was successfully synthesized from poly(ethylene oxide) and DEAEMA. Conductivity measurements showed that the three polymeric surfactants could be switched numerous times via bubbling with CO_2_/N_2_. The surface activity of aqueous solutions of the polymers was controlled by the absence and presence of CO_2_. Emulsions prepared with the CO_2_–switchable copolymers showed reversible stability with addition of CO_2_. Moreover, copolymer **3** displayed switchable viscosity, responding to CO_2_ as a stimulus. On account of its cyclic structure, copolymer **1** had a cloud point of 75 °C. In summary, the three copolymers had different properties because of their unique topologies. This characteristic is helpful for the design of polymeric surfactants for different applications.

## Figures and Tables

**Table 1:** Comparison of the properties of copolymers **1**, **2**, and **3.**

Polymer	Molecular Weight Measured by GPC (Da)	PEO: PDEAEMA^a^	PDI	CMC (%)	The Lowest Surface Tension (mN/m)	Viscosity at 1.5%, 25 °C, w/CO_2_ (mPa·s)	Viscosity at 1.5%,25 °C, w/o CO_2_ (mPa·s)	Cloud Point at 1.0% (°C)
1	2970	100:13	1.22	0.12	37.3	1.5	1.5	75
2	2680	100:11	1.27	0.11	37.5	1.4	1.4	66
3	3120	100:15	1.26	0.12	35.6	1.5	57.2	60

a, The ratio of the polymerization degree between poly (ethylene oxide) and poly (DEAEMA) segments in each polymer.

## Supplementary Materials

The following are available online at https://www.mdpi.com/2073-4360/12/4/984/s1, Figure S1: 1H NMR spectrum of polymer 1. CClD3 was used as solvent, Figure S2: 1H NMR spectrum of polymer 2. CClD3 was used as solvent. Figure S3: 1H NMR spectrum of polymer 3. CClD3 was used as solvent, Figure S4: 1H NMR spectrum of protonated polymer 2 by CO2 and H2O. CD3OD was used as solvent.

## References

[1] Lin S., Theato P. (2013). CO_2_-responsive polymers. Macromol. Rapid Commun..

[2] Yan Q., Wang J., Yin Y., Yuan J. (2013). Breathing polymersomes: CO_2_–tuning membrane permeability for size–selective release, separation, and reaction. Angew. Chem. Int. Ed..

[3] Guo Z.R., Feng Y., He S., Qu M.Z., Chen H.L., Liu H.B., Wu Y.F., Wang Y. (2013). CO_2_-Responsive “smart” single-walled carbon nanotubes. Adv. Mater..

[4] Liu H., Guo Z., He S., Yin H., Fei C., Feng Y. (2014). CO_2_–driven vesicle to micelle regulation of amphiphilic copolymer: Random *versus* block strategy. Polym. Chem..

[5] Liu Y.X., Jessop P.G., Cunningham M., Eckert C.A., Liotta C.L. (2006). Switchable surfactants. Science.

[6] Pinaud J., Kowal E., Cunningham M., Jessop P. (2012). 2–(Diethyl)aminoethyl methacrylate as a CO_2_–switchable comonomer for the preparation of readily coagulated and redispersed polymer latexes. ACS Macro Lett..

[7] Mercer S.M., Jessop P.G. (2010). “Switchable water”: Aqueous solutions of switchable ionic strength. ChemSusChem.

[8] Su X., Jessop P.G., Cunningham M.F. (2012). Surfactant–free polymerization forming switchable latexes that can be aggregated and redispersed by CO_2_ removal and then readdition. Macromolecules.

[9] Su X., Cunningham M.F., Jessop P.G. (2013). Switchable viscosity triggered by CO_2_ using smart worm–like micelles. Chem. Commun..

[10] Zhang Y.M., Feng Y.J., Wang Y.J., Li X.L. (2013). CO_2_–switchable viscoelastic fluids based on a pseudogemini surfactant. Langmuir.

[11] Zhang Y.M., Feng Y., Wang J.Y., He S., Guo Z.R., Chu Z.L., Dreiss C.A. (2013). CO_2_–switchable wormlike micelles. Chem. Commun..

[12] Zhang Y.M., Chu Z.L., Dreiss C.A., Wang Y.J., Fei C.H., Feng Y.J. (2013). Smart wormlike micelles switched by CO_2_ and air. Soft Matter.

[13] Zhou Z., Lu H., Huang Z. (2016). A CO_2_–switchable polymer surfactant copolymerized with DMAEMA and AM as a heavy oil emulsifier. J. Dispers. Sci. Technol..

[14] Raffa P., Wever D.A., Picchioni F., Broekhuis A.A. (2015). Polymeric surfactants: Synthesis, properties, and links to applications. Chem. Rev..

[15] Liu H., Zhao Y., Dreiss C.A., Feng Y. (2014). CO_2_–switchable multi–compartment micelles with segregated corona. Soft Matter.

[16] Honda S., Yamamoto T., Tezuka Y. (2010). Topology–directed control on thermal stability: Micelles formed from linear and cyclized amphiphilic block copolymers. J. Am. Chem. Soc..

[17] Yamagishi A. (2000). Thermophiles and life science in space. Biol. Sci. Space.

[18] Arakawa K., Eguchi T., Kakinuma K. (2001). 36–Membered macrocyclic diether lipid is advantageous for archaea to thrive under the extreme thermal environments. Bull. Chem. Soc. Jpn..

[19] Adachi K., Honda S., Hayashi S., Tezuka Y. (2008). ATRP–RCM synthesis of cyclic diblock copolymers. Macromolecules.

[20] Matyjaszewski K. (2018). Advanced materials by atom transfer radical polymerization. Adv. Mater..

[21] Aoki S., Mikami K., Terada M., Nakai T. (1993). Enantio– and diastereoselective catalysis of addition reaction of allylic silanes and stannanes to glyoxylates by binaphthol–derived titanium complex. Tetrahedron.

[22] Vougioukalakis G.C., Grubbs R.H. (2010). Ruthenium–based heterocyclic carbene–coordinated olefin metathesis catalysts. Chem. Rev..

[23] Kimerling A.S., Rochefort W.E., Bhatia S.R. (2006). Rheology of block polyelectrolyte solutions and gels: A review. Ind. Eng. Chem. Res..

