# Supplementary material for: Diblock, Triblock and Cyclic Amphiphilic Copolymers with CO2 Switchability: Effects of Topology
Source: Polymers (Basel). 2020 Apr 24;12(4):984. doi: 10.3390/polym12040984 (PMC7240586; doi:10.3390/polym12040984)
Supplement: Supplementary file 1 [file polymers-12-00984-s001.pdf]

Supporting Information

Diblock, Triblock and Cyclic Amphiphilic Copolymers with CO_2 Switchability: Effects of Topology

Yuting Jiang [1], Tong Zhang [1], Zheng Yi [2], Yixiu Han [2], Xin Su [1,*] and Yujun Feng [1,*]

[1] Polymer Research Institute, State Key Laboratory of Polymer Materials Engineering, Sichuan University, Chengdu 610065, China; jiangyt99@126.com (Y.J.); zhangtong2099@163.com (T.Z.)

[2] The Second Research Institute of Civil Aviation Administration of China, Chengdu 610041, China; yizheng@caacsri.com (Z.Y.); hanyixiu@caacsri.com (Y.H.)

* Correspondence: xinsu@scu.edu.cn (X.S.); yjfeng@scu.edu.cn (Y.F.); Tel.: +86–28–85408037(X.S&Y.F.)

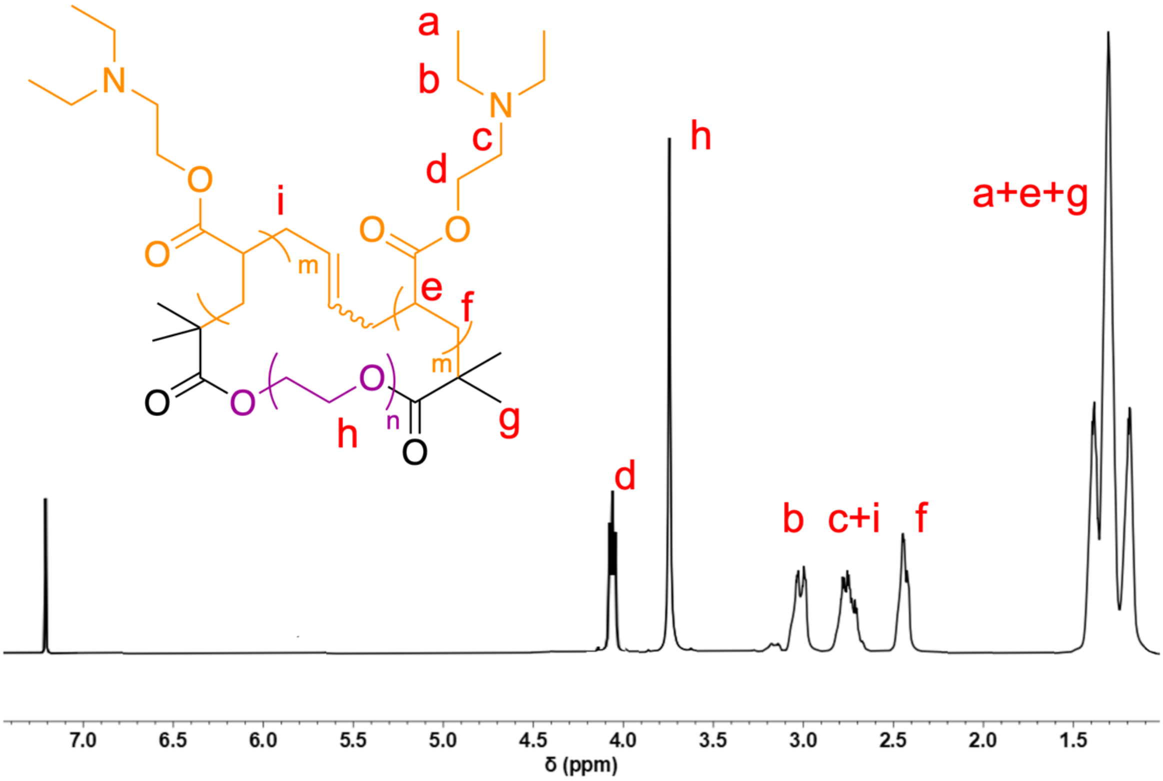

Figure S1. ^{1}H NMR spectrum of polymer 1. $CClD_3$ was used as solvent.

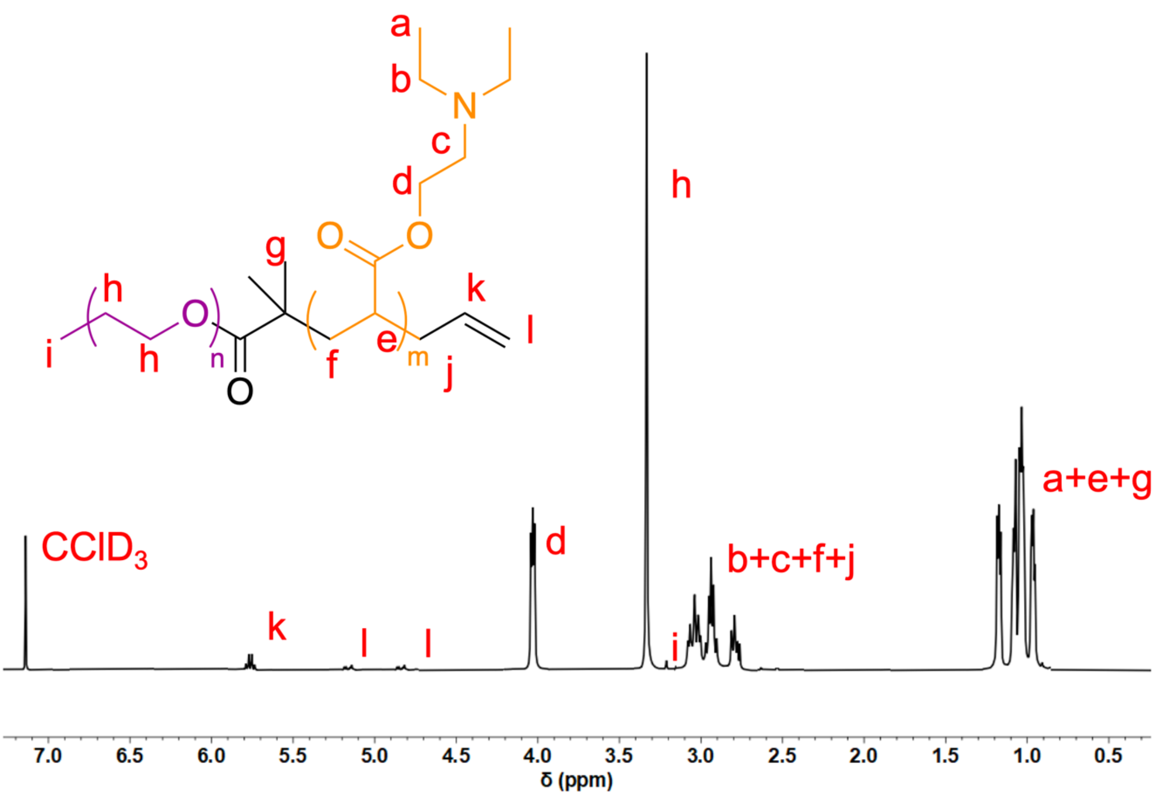

Figure S2. ^{1}H NMR spectrum of polymer 2. $CClD_3$ was used as solvent.

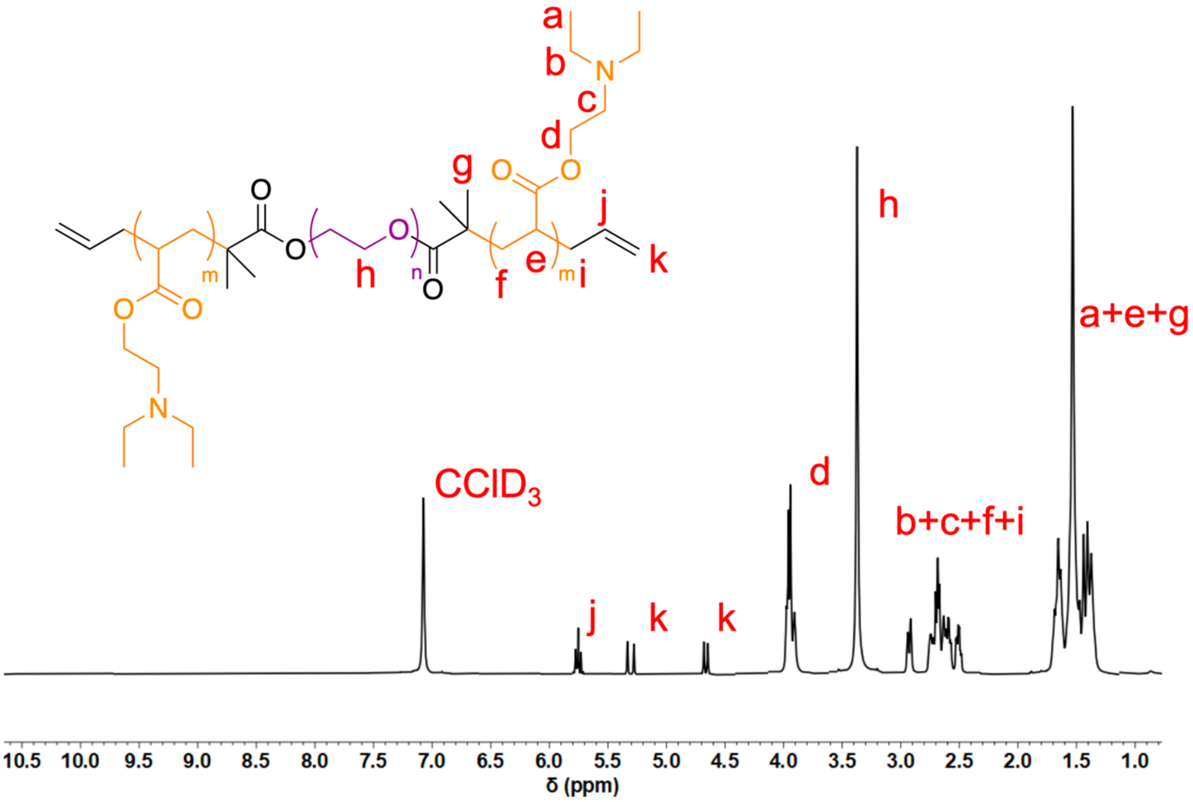

Figure S3. ^{1}H NMR spectrum of polymer 3. $CClD_3$ was used as solvent.

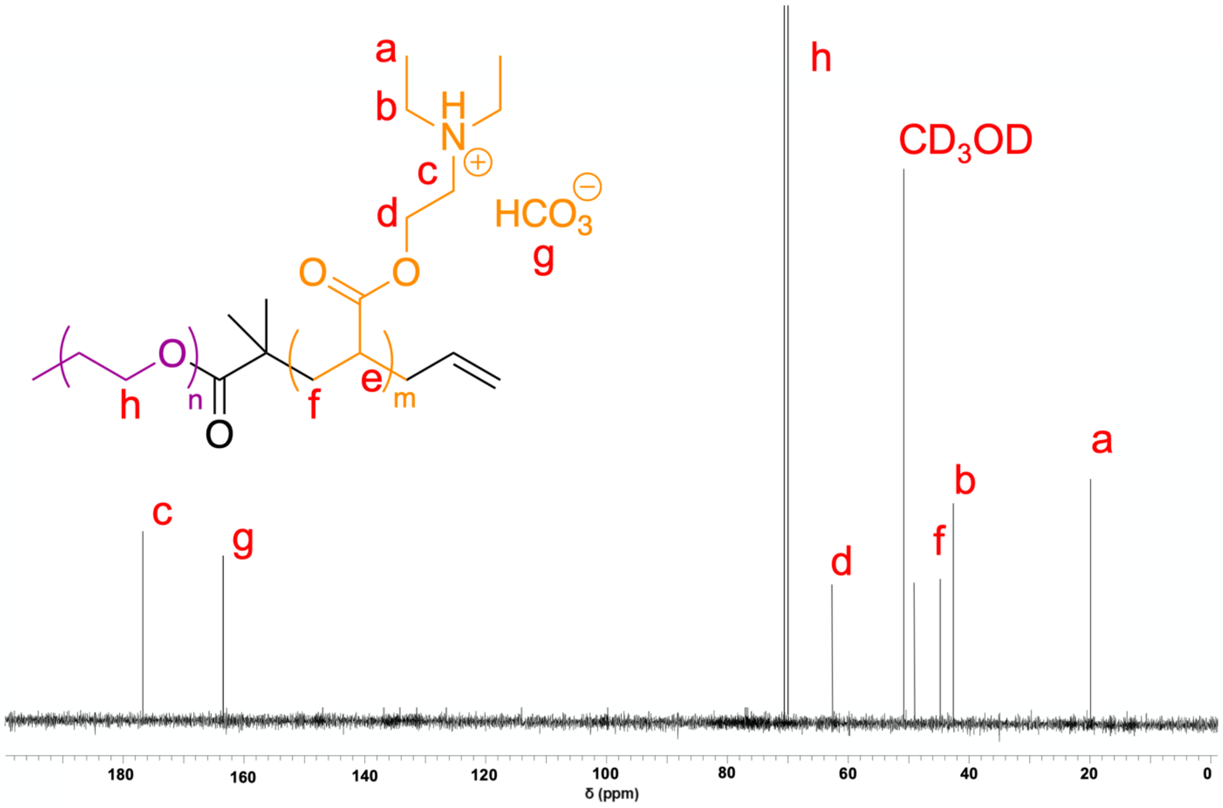

Figure S4. ^{1}H NMR spectrum of protonated polymer 2 by CO_2 and H_2O. CD3OD was used as solvent.
